# Feasibility of a ctDNA multigenic panel for non‐small‐cell lung cancer early detection and disease surveillance

**DOI:** 10.1002/1878-0261.70131

**Published:** 2025-10-10

**Authors:** Giovanna Maria Stanfoca Casagrande, Marcela de Oliveira Silva, Mariana Bisarro dos Reis, Rodrigo de Oliveira Cavagna, Luciane Sussuchi, Icaro Alves Pinto, Natalia Zampieri Pontes, Rodrigo Sampaio Chiarantano, Flavio Augusto Ferreira da Silva, Pedro de Marchi, Letícia Ferro Leal, Rui M. Reis

**Affiliations:** ^1^ Molecular Oncology Research Center, Barretos Cancer Hospital Brazil; ^2^ Barretos School of Health Sciences Dr. Paulo Prata – FACISB Brazil; ^3^ Department of Radiology Barretos Cancer Hospital Brazil; ^4^ Department of Medical Oncology Barretos Cancer Hospital Brazil; ^5^ Oncoclinicas Group Rio de Janeiro Brazil; ^6^ Life and Health Sciences Research Institute (ICVS), School of Medicine University of Minho Braga Portugal

**Keywords:** actionable mutations, ctDNA, liquid biopsy, lung cancer

## Abstract

The detection of actionable mutations in liquid biopsies is a crucial tool for precision oncology in patients with non‐small‐cell lung cancer (NSCLC). We evaluated actionable alterations using a multigene panel in circulating tumor DNA (ctDNA) from Brazilian NSCLC patients. We analyzed 32 samples from 30 patients with NSCLC, including four samples from a lung cancer screening program. ctDNA isolation and library preparation were performed using the Oncomine Lung cfDNA Assay, which covers 11 actionable genes, and sequenced on an Ion S5 Sequencer. The IonReporter 5.20 software was used for variant calling. Median read coverage reached 80 967, with a detection limit of 0.1%. *TP53* (40.6%)*, KRAS* (28.1%), and *EGFR* (12.5%) were the most frequently mutated genes, particularly in patients who had previously received treatment. *BRAF, MAP2K1, PIK3CA,* and *ALK* mutations were observed at lower frequencies (6.2%, 3.1%, 3.1%, and 3.1%, respectively). The *EGFR* p.T790M mutations related to resistance were identified in a patient who had been previously treated, and the *TP53* p.R248Q mutation was discovered in an asymptomatic patient before diagnosis. No variants were observed in *NRAS, ROS1,* and *MET* genes. Our data showed that this commercial NGS panel could detect actionable mutations, enabling early detection, treatment monitoring, and disease surveillance.

## Graphical abstract

Plasma‐based detection of actionable mutations is a promising approach in lung cancer management. Analysis of ctDNA with a multigene NGS panel identified *TP53*, *KRAS*, and *EGFR* as the most frequently altered, with *TP53* and *KRAS* in treatment‐naïve patients and *TP53* and *EGFR* in previously treated patients. These findings highlight liquid biopsy as a feasible strategy enabling faster clinical decision‐making.

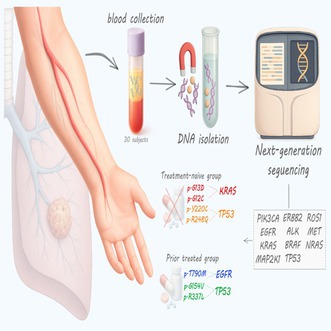

**Abbreviations**
*ALK*
: anaplastic lymphoma kinase
*BRAF*
: v‐raf murine sarcoma viral oncogene homolog B
cfDNA: cell‐free DNA
ctDNA: circulating tumor DNA
*EGFR*
: Epidermal Growth Factor Receptor
*KRAS*
: Kirsten rat sarcoma
LOD: limit of detection
*MAP2K1*
: Mitogen‐activated Protein Kinase Kinase 1
*MET*
: met proto‐oncogene
MMC: Median Molecular Coverage
MRC: Median Read Coverage
NGS: Next‐Generation Sequencing
*NRAS*
: Neuroblastoma RAS Viral Oncogene Homolog
NSCLC: non‐small cell lung cancer
*PIK3CA*
: Phosphatidylinositol‐4,5‐Bisphosphate 3‐Kinase Catalytic Subunit Alpha
PT: prior treated
*ROS1*
: ROS proto‐oncogene 1
SCC: squamous cell carcinoma
TN: treatment naïve
*TP53*
: Tumor Protein p53
UMI: Unique Molecular Identify

## 1 Introduction

Lung cancer is the leading cause of cancer‐related deaths worldwide and in Brazil. Understanding the biology of lung cancer and identifying driver alterations has led to the advent of targeted therapies, which offer significant clinical benefits [1].

A liquid biopsy enables the detection of driver genetic alterations in a noninvasive manner. The detection of circulating cell‐free DNA, (cfDNA), which is released from cells undergoing apoptosis, necrosis, and/or active secretion, and when released from tumor cells, the circulating tumor DNA (ctDNA), is currently used to aid in the diagnosis, prognosis, and treatment of cancer patients [2]. The ctDNA not only overcomes the well‐reported issue of tumor availability in small NSCLC biopsies but also is more representative of tumor heterogeneity than tumor biopsies and can be detected in clinically asymptomatic patients [3].

In this study, we investigate the feasibility of detecting actionable alterations using a commercial lung multigene panel in ctDNA derived from plasma of treatment‐naïve (TN) and prior‐treated (PT) NSCLC patients, as well as participants of a lung cancer screening program of a Brazilian institution.

## 2 Methods

### 2.1 Study design and sample processing

This retrospective study included 30 patients with NSCLC diagnosed between 2008 and 2020 at the Department of Thorax (*n* = 26) and the Lung Cancer Screening Program (*n* = 4) of Barretos Cancer Hospital, Brazil [4]. A total of 32 plasma samples were collected. Twenty‐four were from 22 treatment‐naïve (TN) patients, and eight samples were from prior‐treated (PT) patients. Sociodemographic, clinical, and pathological data were collected from patients' medical records. The study was approved by the Barretos Cancer Hospital IRB (CAAE: 55631316.2.0000.5437; CEP‐HCB: 1139/2016) and adhered to the principles of the Declaration of Helsinki. The requirement for informed consent was waived by the Institutional Review Board due to the retrospective nature of the study and the high mortality rate associated with lung cancer, following the Brazilian regulations (Resolution CNS No. 466/2012 and Law No. 14874/2024). Blood samples were collected on (BD Vacutainer K_2_EDTA^®^) and cell‐free DNA BCT tubes (Streck Corporate, La Vista, NE, USA). Plasma processing involved a centrifuge (16 000 ***g*** for 10 min at 4 °C) prior to cfDNA isolation to remove any blood residues and prevent cell contamination. Plasma samples from Cell‐Free DNA BCT underwent an additional step of lysis using proteinase K (PK).

CfDNA was isolated from 1 to 4 mL of plasma (subject to availability), using the MagMax™ Cell‐free DNA Isolation Kit (Thermo Fisher Scientific, Waltham, MA, USA), and quantified by fluorometry (Qubit^®^ 2.0, dsDNA High Sensitivity Assay, Thermo Fisher Scientific). DNA fragment distribution was evaluated by TapeStation using the Agilent High Sensitivity D1000 ScreenTape Assay kit (Agilent Technologies, Santa Clara, SA, USA).

### 2.2 Ion S5 sequencing and data analysis

Next‐generation sequencing (NGS) was performed in the cfDNA using the commercial Oncomine™ Lung cfDNA Assay, which covers over 150 hotspot regions in 11 driver genes, including *ALK, BRAF, EGFR, ERBB2, KRAS, MAP2K1, MET, NRAS, PIK3CA, ROS1*, and *TP53*.

The sequencing libraries were prepared using an initial 6–30 ng of cfDNA in two rounds of PCRs for library amplification and target selection. Libraries were quantified using the Ion Library Quantification Kit (Life Technologies, Carlsbad, CA, USA) according to the manufacturer's instructions. The final libraries were diluted to 40 pmol, and a pool comprised of 16 sample libraries was employed for template preparation in the IonChef System (Life Technologies) using the Ion 540™ chip (Life Technologies). The run quality assessment considered the following parameters acceptable: > 60% chip loading, total read count between 60 and 80 million, < 45% polyclonality.

Raw sequencing data were processed in the Ion S5™ Torrent Server, and the reads were aligned to the hg19 human reference genome using the Torrent Suite™ Software (Thermo Fisher Scientific). Variant calling and annotation were performed by the IonReporter IR 5.20 software, following the Oncomine Lung Liquid Biopsy ‐ w1.9 ‐ DNA ‐ Single Sample (version 5.20) workflow. The data that support the findings of this study are available in the European Genome‐phenome Archive and are accessible through https://ega‐archive.org/datasets/EGAD50000000908, EGA Series accession number [EGAD50000000908].

## 3 Results

### 3.1 Patient's clinicopathological features

Table 1 summarizes the clinicopathological characteristics of patients. The median age of the cohort was 64 years (min: 19; max: 89), and half of the patients were current smokers (*n* = 15) (Table 1). Forty percent of the patients (*n* = 12) were diagnosed with adenocarcinomas, 36.6% (*n* = 11) were classified as squamous cell carcinomas, and 23.4% were considered as NSCLC with no other specifications (NSCLC NOS; *n* = 7) (Table 1). Most patients are presented with nonmetastatic disease (Table 1). Plasma samples were mainly from treatment‐naïve patients (24 samples from 22 patients). For prior‐treated patients (*n* = 8), treatment regimens are described in Table S1.

**Table 1:** Clinicopathological features of NSCLC patients (*n* = 30). Disease stage was determined according to the AJCC 7th edition. NSCLC NOS, non‐small cell lung cancer with no other specifications.

	All patients (*n* = 30)
Median Age (min ‐ max)	64 (19–89)
Sex	
Male	17 (56.6%)
Female	13 (43.4%)
Smoking status	
Current	15 (50%)
Quitter	11 (36.6%)
Never	3 (10%)
No information	1 (3.4%)
Histology	
Adenocarcinoma	12 (40%)
Squamous Cell Carcinoma	11 (36.6%)
NSCLC NOS	7 (23.4%)
Disease Stage	
I	4 (13.4%)
II	4 (13.4%)
III	11 (36.6%)
IV	11 (36.6%)

### 3.2 Assay quality control and data analysis

Fragment analysis of cfDNA isolated from plasma showed the presence of fragments ranging from 170 to 400 base pairs in length, which is consistent with the presence of ctDNA (Fig. S1). The average of cfDNA used for library preparation was 21.13 ng (min = 6.07 ng; max = 181.20 ng; Table 2).

**Table 2:** Detected mutations in plasma ctDNA. cfDNA, cell‐free DNA; ND, not detected; NGS, Next‐Generation Sequencing; PT, Prior Treatment; TN, Treatment naïve; VAF, Variant Allele Frequency.

Patient	Quantification (ng·uL^−1^)	Total amount of cfDNA (ng)	Amount of cfDNA per mL of plasma (ng·uL^−1^)	Plasma volume per sample (mL)	Input cfDNA for NGS (ng)	Limit of detection (%; median)	Time collection	cfDNA			
								Gene	Variant	VAF (%)	Variant type
AD_35	1.03	15.45	0.69	1.5	13.39	0.15	TN	*MAP2K1*	p.P124Q	14.70	Missense
AD_36	1.02	15.30	0.73	1.4	9.18	0.12	PT	*TP53*	p.R337L	0.13	Missense
AD_70	1.01	15.15	0.67	1.5	12.12	0.10	TN	*ALK*	p.R1275Q	0.06	Missense
								*TP53*	p.R175H	0.14	Missense
AD_82	1.58	23.70	1.05	1.5	12.00	0.13	PT	*TP53*	p.G154V	7.10	Missense
AD_91	3.42	51.30	2.28	1.5	20.00	0.06	TN	*TP53*	p.Y220C	0.09	Missense
AD_92	1.33	19.95	1.02	1.3	10.84	0.12	TN	*KRAS*	p.Q61H	0.07	Missense
								*KRAS*	p.G12V	0.15	Missense
								*TP53*	p.G245C	8.11	Missense
AD_105	0.72	10.80	0.72	1.00	12.08	0.10	PT	*EGFR*	p.E709K	0.56	Missense
								*BRAF*	p.G469A	0.06	Missense
								*TP53*	p.G245V	0.13	Missense
								*TP53*	p.Y220C	0.08	Missense
AD_130	2.18	32.70	1.09	2.00	20.00	0.06	PT	ND			
AD_140	0.82	12.30	0.59	1.4	14.63	0.10	TN	ND			
AD_142	1.01	15.15	1.01	1.00	8.36	0.18	TN	*KRAS*	p.G12C	9.86	Missense
AD_150	12.3	184.50	6.15	2.00	25.00	0.06	TN	*KRAS*	p.G12C	18.40	Missense
								*TP53*	p.G154V	22.33	Missense
AD_159	3.24	48.60	1.62	2.00	20.00	0.08	TN	*TP53*	p.R282W	0.08	Missense
AD_168	1.85	27.75	1.85	1.00	20.00	0.64	TN	ND			
AD_174	3.18	47.70	3.18	1.00	20.00	0.06	TN	*PIK3CA*	p.E542K	0.30	Missense
								*KRAS*	p.G12C	1.15	Missense
								*TP53*	p.R249S	0.05	missense
AD_177	1.08	16.20	0.60	1.8	8.15	0.19	TN	*KRAS*	p.G13D	0.47	Missense
AD_179	2.98	44.70	1.49	2.00	20.00	0.06	TN	*KRAS*	p.G12V	11.51	Missense
AD_181	0.86	12.90	0.61	1.4	9.22	0.08	TN	*BRAF*	p.G466E	0.17	Missense
AD_205	2.28	34.20	1.52	1.5	20.00	0.08	PT	*EGFR*	p.T790M	0.08	Missense
								*KRAS*	p.G12V	0.06	Missense
								*TP53*	p.R273C	0.05	Missense
								*TP53*	p.H214R	48.25	Missense
AD_216	1.78	26.70	0.99	1.8	12.46	0.11	PT	*KRAS*	p.G12V	0.32	Missense
								*TP53*	p.V157F	0.22	Missense
AD_220	1.94	29.10	1.29	1.5	16.49	0.06	TN	ND			
AD_221	1.09	16.35	0.73	1.5	5.72	0.29	TN	ND			
AD_223	15.1	226.50	10.79	1.4	30.00	0.06	TN	ND			
AD_232	3.32	49.80	1.11	3.00	20.02	0.06	PT	*KRAS*	p.G12D	0.23	Missense
AD_235	1.67	25.05	0.42	4.00	20.04	0.06	PT	*EGFR*	p.E746_A750	0.35	Indel
AD_276	1.66	24.90	0.55	3.00	19.92	0.07	TN	ND			
AD_280	1.43	21.45	0.41	3.5	17.16	0.09	TN	ND			
LS_4	0.6	9.00	0.25	2.4	6.52	0.20	TN	ND			
LS_1	1.15	17.25	0.29	4.00	10.93	0.13	TN	*EGFR*	p.E709K	0.38	Missense
LS_2*	1.06	15.90	0.28	3.8	9.18	0.11	TN	*TP53*	p.R248Q	0.07	Missense
LS_2**	1.39	20.85	0.37	3.8	7.31	0.11	TN	*TP53*	p.Y220C	0.17	Missense
LS_3*	0.92	13.80	0.24	3.8	9.01	0.16	TN	ND			
LS_3**	0.69	10.35	0.18	3.8	12.51	0.14	TN	ND			

The quality parameters of the sequencing runs, considering chip loading, polyclonal percentage, average read length, and the percentage of aligned reads to the reference genome, were within the quality standards determined by the manufacturer (Table S2; Fig. S2). The average of molecular median coverage (MMC) was 3099.40 (SD: 1537.49; range: 465–6943), and the average of molecular read coverage (MRC) of the sequencing runs of cfDNAs was 80 967.43 (SD: 37 885.14; range: 33 456–18 7502) (Table S2; Fig. S3).

### 3.3 cfDNA mutational profile

**Fig. 1:**
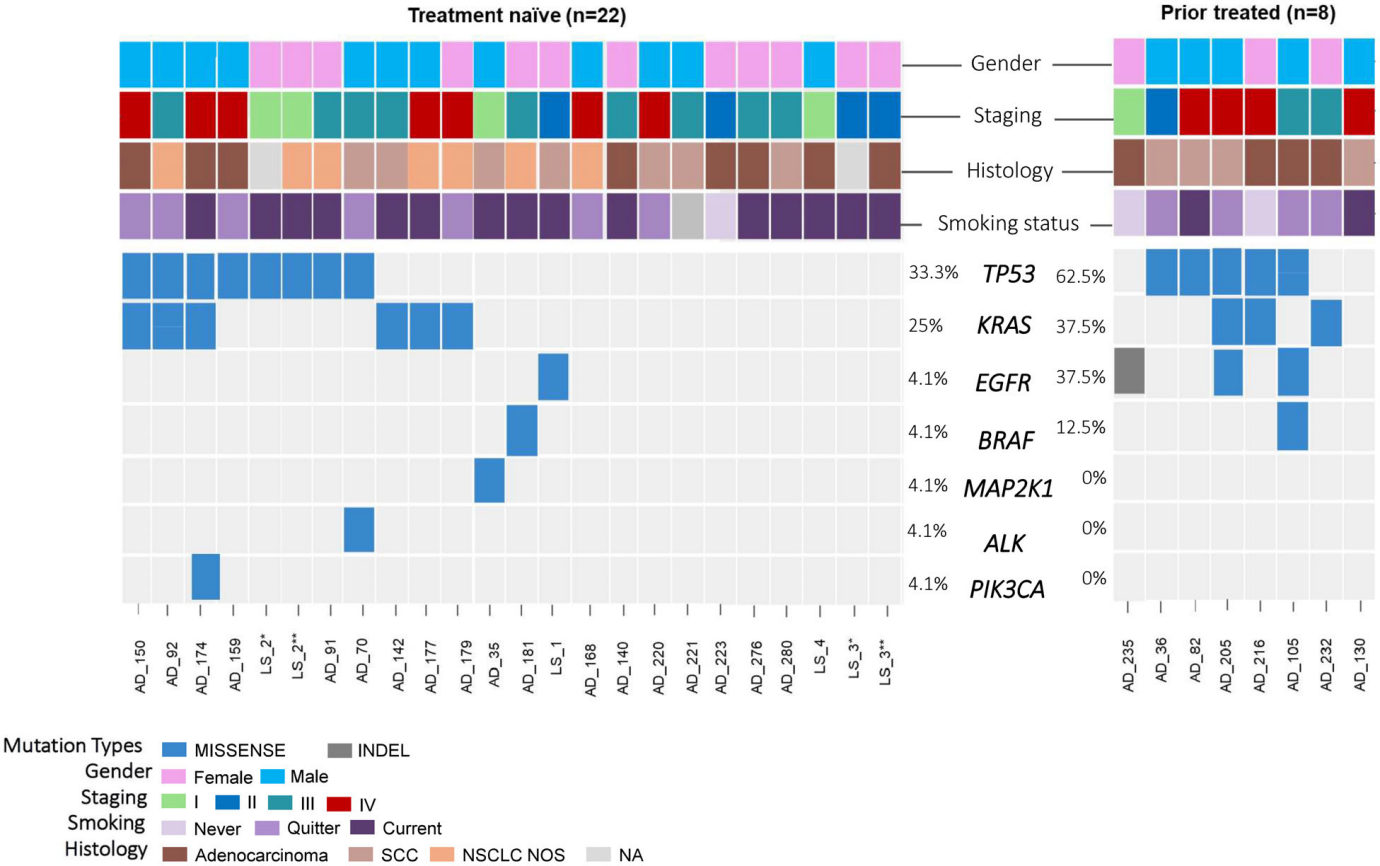
Summary of patient characteristics and genetic mutations in ctDNA samples from treatment naïve patients (*n* = 22; 24 samples) and prior treated patients (*n* = 8). Samples were classified by staging, histology, smoking status, and treatment‐related time of collection. Regarding the type of genetic alteration, they are classified as: missense mutation (blue square) and indels (gray square) and no mutation detected (light gray square). Each column represents a patient. LS_1, LS_2, LS_3, and LS_4 are patients included through the Lung Cancer Screening Program. *first blood collection; **second blood collection, NA: not applicable; LS2* (LungRADS 4a), LS3* (LungRADS 4a).

**Fig. 2:**
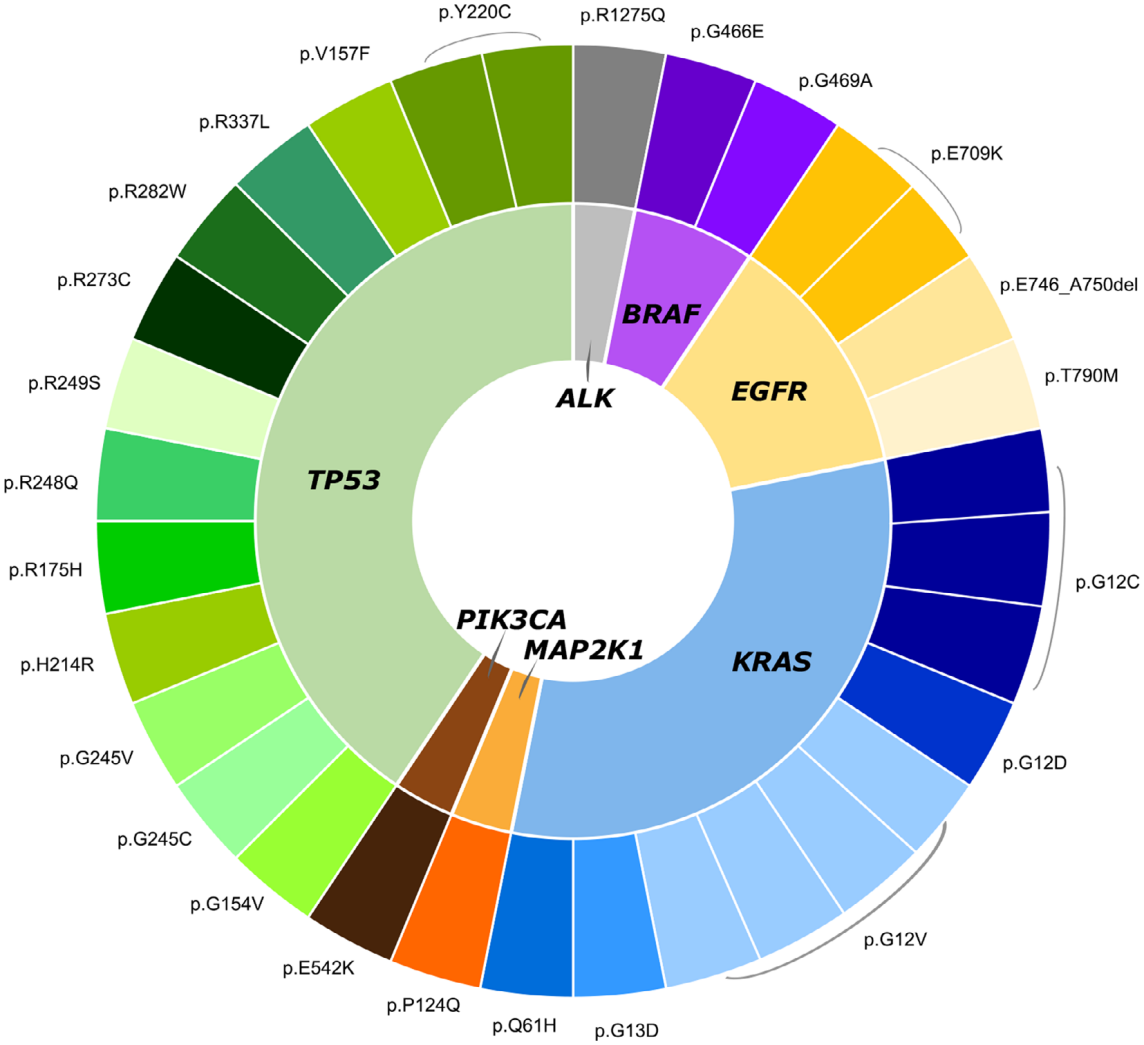
Genes and variants detected in plasma ctDNA analysis. Schematic representation of the 38 variants detected in the liquid biopsy, distributed in seven out of the 11 evaluated genes (*ALK, BRAF, EGFR, ERBB2, KRAS, MAP2K1, MET, NRAS, PIK3CA, ROS1*, and *TP53*). *Variants detected only in prior‐treated patients.

Among the 32 samples evaluated, we found cfDNA mutations in 21 (65.6%) cases, with a higher frequency in the PT group (7/8, 87.5%) (Fig. 1). *TP53* was the most mutated gene (40.6%), with 12 distinct *TP53* variants, and with a higher frequency in the PT than in the TN group (Figs 1 and 2).

The *KRAS* gene had the second highest mutational frequency [25% (*n* = 6) for TN patients; 37.5% (*n* = 3) for PT patients], exhibiting five distinctive variants (Figs 1 and 2). The *EGFR* gene was mutated in one sample of the TN group (4%), and in three samples (37.5%) of the PT group (Figs 1 and 2). Alterations were also observed in the *BRAF* (non‐V600 variants), *MAP2K1, ALK*, and *PIK3CA* genes (Figs 1 and 2). The *TP53* variants co‐occurred with *KRAS, EGFR, BRAF*, and *ALK* variants (Fig. 1). No mutations were observed in the genes *NRAS*, *ROS1*, and *MET*.

We evaluated plasma ctDNA from four patients enrolled in the Lung Cancer Screening Program [4]. One participant (LS_2) presented a “likely pathogenic” *TP53* variant, p.(Arg248Gln), detected at baseline CT screening (Lung‐RADS category 4), despite being asymptomatic and presenting no radiological or clinical evidence of malignancy (Fig. 1). Six months later, during the follow‐up CT screening, the participant was diagnosed with lung cancer.

We next assessed the association between survival time and the presence of detectable ctDNA variants. Although patients with detectable ctDNA variants (ctDNA‐positive) had a shorter median survival (3.7 months) compared to those without detectable variants (ctDNA‐negative; 23.5 months), this difference was not statistically significant (*P* = 0.122; Fig. S4).

## 4 Discussion

The present study demonstrates the feasibility of using a commercial multigene panel to detect actionable mutations in circulating tumor DNA (ctDNA) from plasma samples of both treatment‐naïve individuals, including asymptomatic participants diagnosed through a CT‐based lung cancer screening program, and previously treated NSCLC patients from Barretos Cancer Hospital (Hospital de Amor), Brazil. The ability to identify variants in genes such as *EGFR, KRAS, BRAF*, and *ALK* genes using a single liquid biopsy assay highlights the potential of this approach for early detection and personalized management of NSCLC within the precision oncology [5].

NGS remains the most widely adopted strategy for mutation detection in liquid biopsy. However, the typically low allele frequency of ctDNA variants poses a significant challenge, increasing the risk of false‐negative results. Additionally, liquid biopsy samples require substantially deeper sequencing read depth than tissue‐based analysis to mitigate the impact of random sequencing errors. To address this issue, we employed the technology called Tag sequencing, functionally equivalent to the use of unique molecular identifiers (UMI), which enables highly accurate detection of rare variants [5]. In our study, the quality control metrics showed a high median read coverage with a detection limit as low as 0.1%, further underscoring the sensitivity of this multigenic panel for detecting low‐frequency mutations in ctDNA.

In the present study, we identified *TP53* and *KRAS* as the most frequently mutated genes in ctDNA, consistent with previous reports on tumor tissue from Brazil, underscoring their role in NSCLC pathogenesis [6]. We identified *EGFR* ctDNA mutations in 4% of treatment naïve and 37% of prior‐treated samples. *EGFR* mutations usually occur in 10–30% of Caucasian and admixed patients and approximately in 50% of Asian patients with NSCLC [7]. *EGFR* mutational frequencies may also vary depending on the detection method (e.g., single‐gene assays vs. NGS panels), particularly because some widely used commercial tests have failed to identify rare *EGFR* variants, such as exon 20 insertions and the p.E709K mutation. These variants have been previously reported using Sanger sequencing and NGS, respectively [7, 8]. This highlights the importance of understanding the mutational profile of each population to inform the most appropriate testing strategies for routine settings.

Somatic mutations in the *MAP2K1* and *PIK3CA* genes have not been often observed in NSCLC patients [1]. In the present series, this frequency is moderately higher and might be influenced by tumor clonality and the treatment strategy applied to these cases. The absence of mutations in the *NRAS*, *ROS1*, and *MET* genes is expected, given the low mutation frequency of these genes in NSCLC.

Identifying *EGFR* mutations primarily in prior‐treated samples may reflect acquired resistance to EGFR inhibitors, a well‐documented phenomenon in NSCLC [9]. As expected, the case harboring the *EGFR* p.(T790M) mutation was previously treated with dacomitinib, consistent with the emergence of acquired resistance mechanisms [10]. The frequency of *KRAS* mutations observed in our cohort was 18%, notably higher than reported in European populations. To our knowledge, there are no published data on *KRAS* mutation status assessed by liquid biopsy for squamous cell lung carcinoma among admixed Brazilian patients, limiting direct comparison with our findings. Additionally, *KRAS* mutations are often associated with later stages of tumor evolution and tend to occur in subclonal populations, which may impact their detectability and clinical interpretation [11]. Although *TP53* is not an actionable gene, it is associated with disease outcomes [6]. Thus, the multiplex detection of *EGFR*, *KRAS*, and *TP53* variants in minimally invasive samples can play an adjuvant role in the clinical management of NSCLC patients.

Importantly, we explored the potential utility of ctDNA plasma in the context of CT‐based lung cancer screening for early cancer diagnosis. Notably, two of four asymptomatic participants exhibited detectable driver mutations in plasma ctDNA prior to radiological and pathological confirmation of disease, highlighting the value of these biomarkers for early identification and monitoring of high‐risk individuals.

Despite the promising results, our study presents some limitations. No ctDNA mutations were identified in eight patients, half of whom were diagnosed with earlier stages. This may be attributable to a lower tumor mutation burden and technical challenges associated with detecting ctDNA in early‐stage cancers [4]. Moreover, the commercial panel employed was primarily designed for lung adenocarcinomas and encompasses a limited set of driver genes, does not capture RNA‐based alterations, such as *ALK*, *RET*, *ROS1* gene fusions, and exon skipping events, which are clinically actionable variants in lung adenocarcinoma. Another issue is the small sample size, particularly in the prior‐treated subgroup, which may affect the statistical power and generalizability of our findings. Furthermore, while the detection of somatic mutations in plasma ctDNA holds great potential for noninvasive cancer diagnostics, the confounding impact of clonal hematopoiesis of indeterminate potential (CHIP) must be considered. Indeed, the role of CHIP in interpreting liquid biopsy results has been increasingly recognized in recent literature. However, at the time this panel was developed and implemented, CHIP‐specific filtering strategies were not yet standard practice in clinical ctDNA testing. The panel itself was not optimized to differentiate CHIP‐associated variants, and no matched germline DNA was sequenced to support variant origin classification. This may have led to the inadvertent inclusion of CHIP‐related mutations, which could potentially impact the specificity of our findings. Therefore, future studies should aim to incorporate larger and more diverse patient cohorts, utilize broader sequencing panels, including RNA‐based assays, and integrate germline DNA analysis and CHIP‐aware filtering pipelines to enhance the accuracy and clinical utility of ctDNA profiling.

## 5 Conclusion

Our study confirms the feasibility of using a plasma‐based ctDNA multigene panel to detect clinically actionable mutations in both treatment‐naïve and prior‐treated NSCLC patients. Liquid biopsies thus represent a minimally invasive, time‐efficient tool that may complement existing diagnostic modalities, supporting early detection and NSCLC management.

## Conflict of interest

The authors declare no conflict of interest.

## Author contributions

G.M.S.C., M.O.S., and I.A.P. wrote the main manuscript text. M.O.S., M.B.R., N.Z.P., and L.F.L. conducted the experiments. N.Z.P., F.A.F.S., R.C.S., and P.M. collected clinical data. G.M.S.C., M.O.S., L.S., and R.O.C. performed the statistical analyses. G.M.S.C., R.O.C., L.S., L.F.L., and R.M.R. contributed to the interpretation of the results. R.C.S., R.M.R., and L.F.L. conceived the study, secured funding, and were responsible for its overall direction and planning. All authors reviewed and approved the final manuscript.

## Supporting information


**Fig. S1.** Representative graphs of fragment analysis of plasma ctDNA samples (between 170 and 400 bp) using TapeStation (Agilent Technologies, Santa Clara, SA, USA).

**Fig. S2.** Quality control of the NGS experiments.

**Fig. S3.** Read counts of all amplicons analyzed in the Oncomine cfDNA lung assay.

**Fig. S4.** Overall survival of patients with ctDNA‐positive and ctDNA‐negative.

**Table S1.** Overview of treatments administered by a subset of pretreated patients prior to plasma collection.
**Table S2.** Sequencing analysis parameters of the samples.

## Data Availability

The data that support the findings of this study are available upon request from the corresponding author. The data are not publicly available due to privacy or ethical restrictions. The genomic data that support the findings of this study are openly available in the European Genome‐phenome Archive and ega‐archive.org/datasets/EGAD50000000908, reference number [EGAD50000000908].
