# Supplementary figures and images for: Feasibility of a ctDNA multigenic panel for non‐small‐cell lung cancer early detection and disease surveillance
Source: Mol Oncol. 2025 Oct 10;20(3):629–36. doi: 10.1002/1878-0261.70131 (PMC13042580; doi:10.1002/1878-0261.70131)

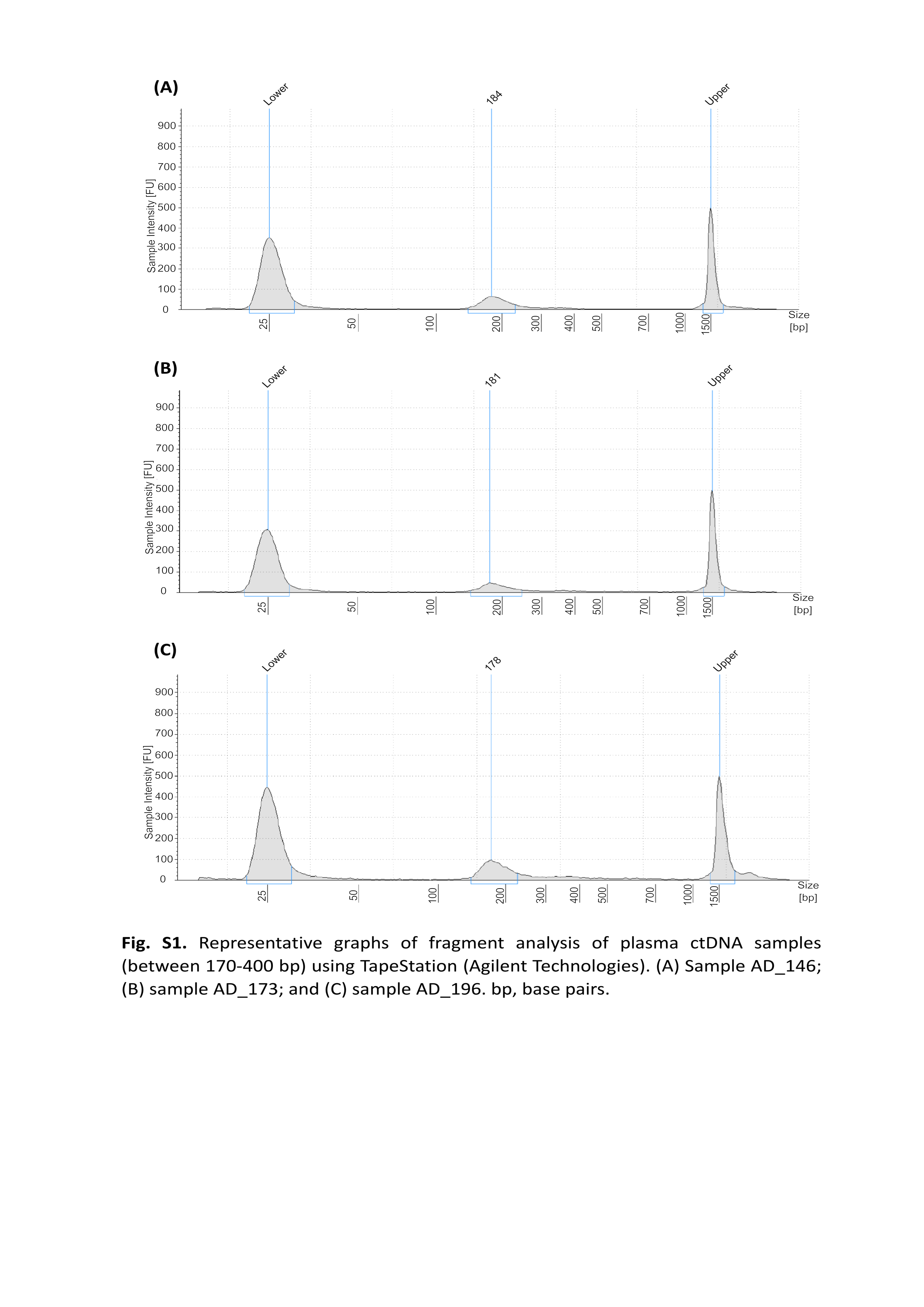

Supplement: Supplementary file 1 — Fig. S1. Representative graphs of fragment analysis of plasma ctDNA samples (between 170 and 400 bp) using TapeStation (Agilent Technologies, Santa Clara, SA, USA). [file MOL2-20-629-s002.png]
