# Supplementary material for: Feasibility of a ctDNA multigenic panel for non‐small‐cell lung cancer early detection and disease surveillance
Source: Mol Oncol. 2025 Oct 10;20(3):629–36. doi: 10.1002/1878-0261.70131 (PMC13042580; doi:10.1002/1878-0261.70131)
Supplement: Supplementary file 2 — Fig. S2. Quality control of the NGS experiments. [file MOL2-20-629-s005.pdf]

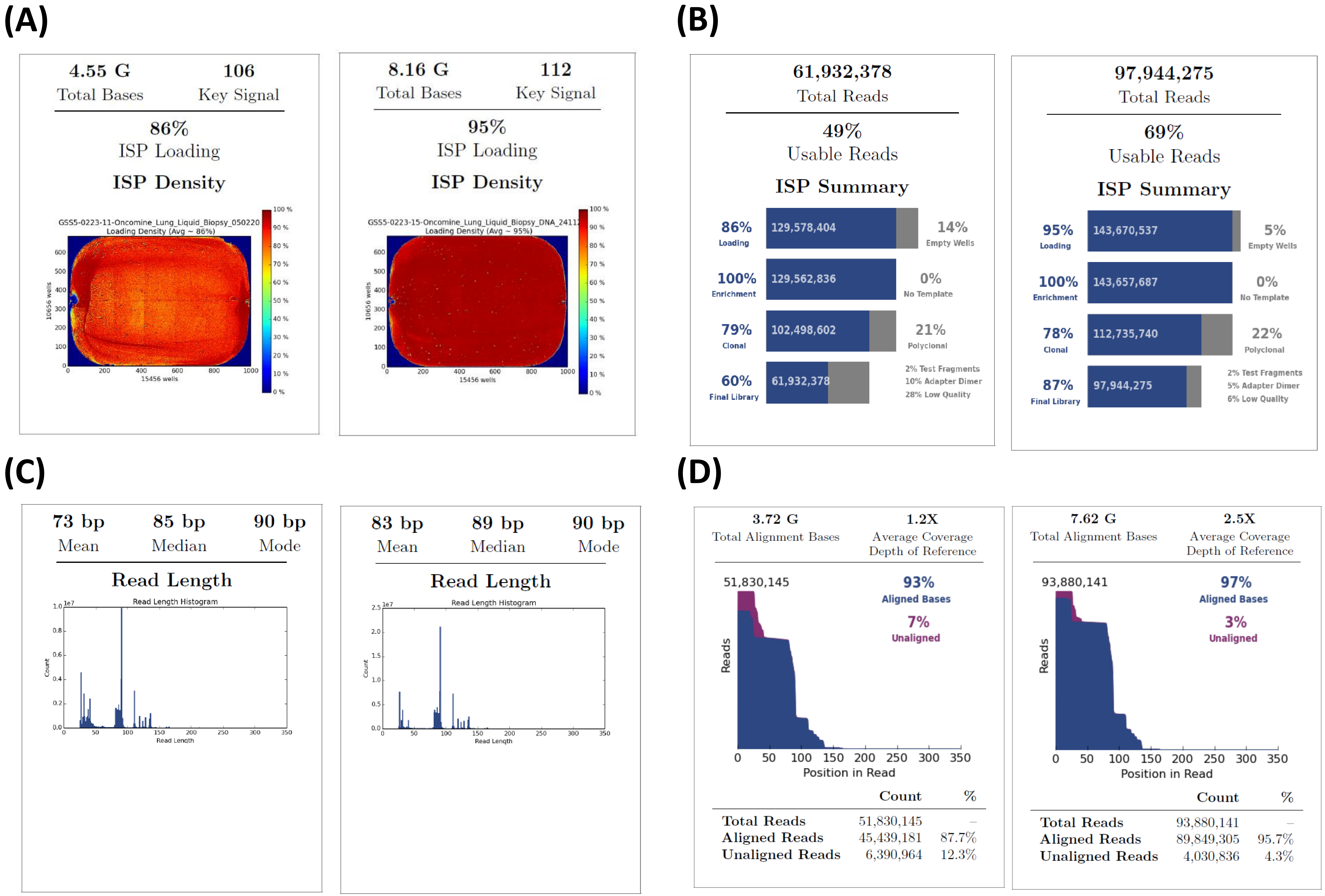

Fig. S2. Quality control of the NGS experiments. Representing the two NGS runs performed (16 samples/each). (A) Loading of the chip (Ion 540™ Chip), considered suitable greater than 60%, and loading reaching 86 and 95%. (B) Summary of ISPs, including chip loading, enrichment, polyclonally, and percentage of libraries referring to valid reads. (C) Length of reads obtained, on average 85 and 80bp. (d) Distribution of valid reads and percentage of alignment of the bases (93 and 97%) with the reference genome (hg19). ISP, Ion Sphere particle; bp, base pairs.
