# Supplementary material for: Feasibility of a ctDNA multigenic panel for non‐small‐cell lung cancer early detection and disease surveillance
Source: Mol Oncol. 2025 Oct 10;20(3):629–36. doi: 10.1002/1878-0261.70131 (PMC13042580; doi:10.1002/1878-0261.70131)

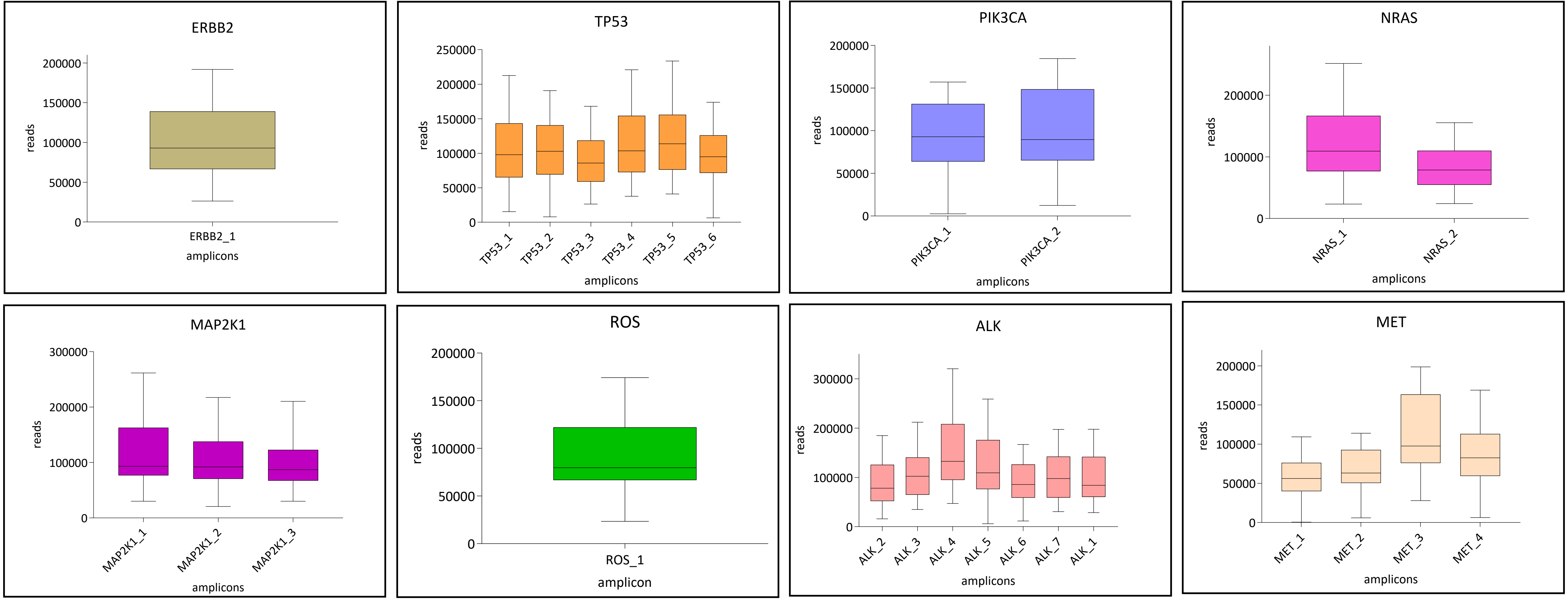

Fig. S3. Read counts of all amplicons analyzed in the Oncomine cfDNA lung assay. Each color represents a different gene; bars represents max and min counts.

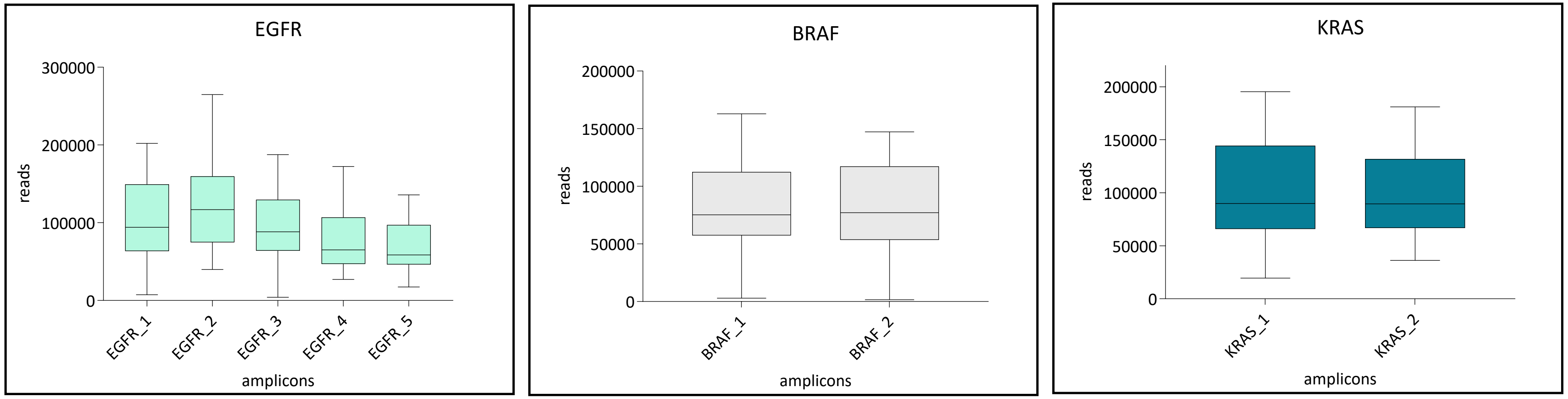

Supplement: Supplementary file 3 — Fig. S3. Read counts of all amplicons analyzed in the Oncomine cfDNA lung assay. [file MOL2-20-629-s001.pdf]
