# Supplementary material for: Feasibility of a ctDNA multigenic panel for non‐small‐cell lung cancer early detection and disease surveillance
Source: Mol Oncol. 2025 Oct 10;20(3):629–36. doi: 10.1002/1878-0261.70131 (PMC13042580; doi:10.1002/1878-0261.70131)
Supplement: Supplementary file 4 — Fig. S4. Overall survival of patients with ctDNA‐positive and ctDNA‐negative. [file MOL2-20-629-s003.pdf]

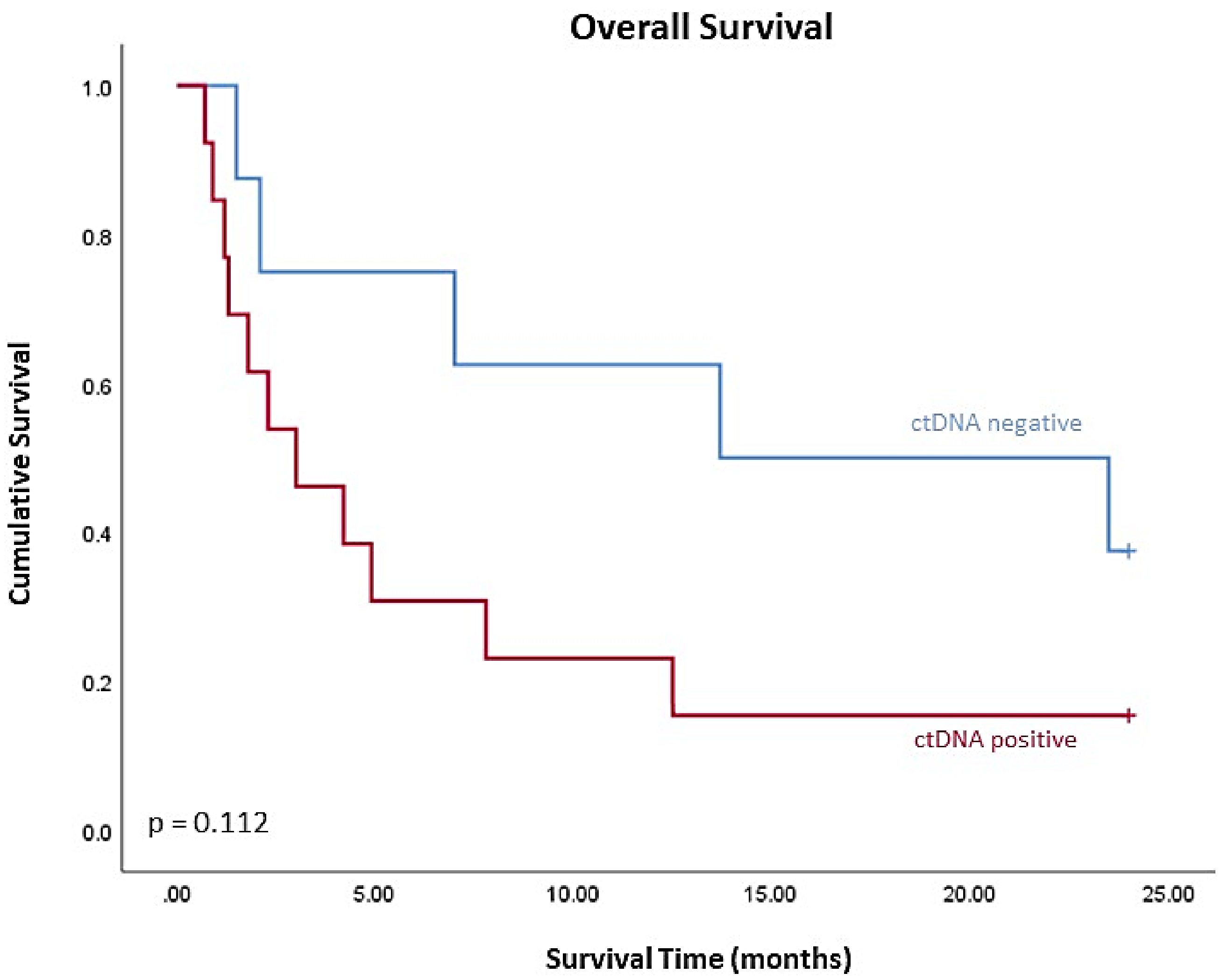

Fig S4. Overall survival of patients with ctDNA-positive and ctDNA-negative. ctDNA-positive was considered when one variant or were detected in plasma; ctDNA-negative was considered when no variant was detected in plasma (p = 0.0112; Log-rank test).
